# A Computational Approach for the Assessment of Executive Functions in Patients with Obsessive–Compulsive Disorder

**DOI:** 10.3390/jcm8111975

**Published:** 2019-11-14

**Authors:** Elisa Pedroli, Filippo La Paglia, Pietro Cipresso, Caterina La Cascia, Giuseppe Riva, Daniele La Barbera

**Affiliations:** 1 Applied Technology for Neuro-Psychology Lab, IRCCS Istituto Auxologico Italiano, Via Magnasco 2, 20149 Milano, Italygiuseppe.riva@unicatt.it (G.R.); 2 Faculty of Psychology, eCampus University, Via Isimbardi, 10, 22060 Novedrate, Italy; 3 Department of Experimental Biomedicine and Clinical Neuroscience, University of Palermo, Piazza Marina, 61, 90133 Palermo, Italy; filippo.lapaglia@unipa.it (F.L.P.); erika.lacascia@unipa.it (C.L.C.); daniele.labarbera@unipa.it (D.L.B.); 4 Psychology Department, Catholic University of Milan, Largo Gemelli 1, 20123 Milan, Italy

**Keywords:** Obsessive–compulsive disorders, virtual reality, multiple errands test, cognitive assessment, executive functions, computational models, decision tree, cross-validation

## Abstract

Previous studies on obsessive–compulsive disorder (OCD) showed impairments in executive domains, particularly in cognitive inhibition. In this perspective, the use of virtual reality showed huge potential in the assessment of executive functions; however, unfortunately, to date, no study on the assessment of these patients took advantage of the use of virtual environments. One of the main problems faced within assessment protocols is the use of a limited number of variables and tools when tailoring a personalized program. The main aim of this study was to provide a heuristic decision tree for the future development of tailored assessment protocols. To this purpose, we conducted a study that involved 58 participants (29 OCD patients and 29 controls) to collect both classic neuropsychological data and precise data based on a validated protocol in virtual reality for the assessment of executive functions, namely, the VMET (virtual multiple errands test). In order to provide clear indications for working on executive functions with these patients, we carried out a cross-validation based on three learning algorithms and computationally defined two decision trees. We found that, by using three neuropsychological tests and two VMET scores, it was possible to discriminate OCD patients from controls, opening a novel scenario for future assessment protocols based on virtual reality and computational techniques.

## 1. Introduction

According to the Diagnostic and Statistical Manual of Mental Disorders, Fifth Edition (DSM-5) [1], patients with obsessive–compulsive disorder (OCD) usually show obsessions and/or compulsions that reduce quality of life because of interference with daily routines, as well as work, social, or family life. This disorder affects about 2% of the population, and the World Health Organization highlighted that OCD is one of the 20 causes of disability in subjects within the 15–44 age range [2]. Moreover, OCD patients show dysfunctions in executive domains, particularly in cognitive inhibition [3], probably caused by a serotoninergic and dopaminergic dysfunction [4]. A deficit in executive functions may give problems when responding to both internal and external requirements, by inhibiting the ability to manage and orient the necessary cognitive resources. Specifically, the term “executive function” indicates a complex domain that includes a large number of cognitive processes and behavioral capabilities such as problem-solving, planning, sequencing, ability to sustain attention, resisting interference, utilizing feedback, cognitive inhibition, multitasking, cognitive flexibility, etc. [5,6,7]. Despite this abnormality, research on neuropsychological impairments in OCD produced unclear results [8]. Analyzing this specific syndrome, we can find some symptoms strongly related to dysexecutive deficits, such as checking behaviors. It is important to understand which one has a causal role in OCD and which one is a consequence of the syndrome [9]. Some articles showed deficits in planning abilities and nonverbal memory, while other studies reported deficits in cognitive flexibility and inhibition, and others displayed no neuropsychological deficits [10,11,12,13,14]. There are many possible explanations, e.g., differences in the methodology, in the instruments used, or in the characteristics of the samples. Specifically, the diversity of tools used during the assessment phase is related to an unsolved debate: the difference between paper-and-pencil and ecological tests [15]. Classic tasks in a classic setting analyze single aspects of complex domains and request simple responses to single events. Conversely, tasks in naturalistic settings may analyze cognitive functions in a complete way, requiring complex answers and, sometimes, the inhibition of inappropriate or irrelevant actions within several subtasks [6]. Therefore, it is critical to increase the ecological validity of a neuropsychological battery, especially for a complex cognitive domain such as an executive function. The assessment procedure has to become more sensitive to different aspects of patient behavior reflecting real-life situations [16]. However, it is too difficult to create a feasible assessment of executive functions during real-life situations because of implementation problems and the difficulty in involving patients in the procedure [17].

Virtual reality (VR) represents a valid solution to address the problems of classical assessment protocols. VR is a new technology that allows users to actively interact in a computer-generated tridimensional environment that simulates the real world [18]. This technology allows subjects to explore and manage several situations inspired by daily experiences using real correspondent behaviors in a more controlled, safe, and low-cost setting than real-life situations [19]. In the last few years, VR was applied for the assessment and rehabilitation of several psychological diseases such as post-traumatic stress disorder [20,21,22], anxiety [23,24], and eating disorders [25], as well as for neuropsychological domains such as neglect [26,27], executive functions [28,29], decision-making [30], spatial memory and orientation [31,32,33], and cognitive rehabilitation of schizophrenia [34].

In the current study, we proposed a computational approach based on classification learning algorithms to discriminate OCD patients from a control group. There were many studies that highlighted the usefulness of a VR-based approach for the assessment of executive functions in OCD patients [35,36,37]. These patients showed a specific pattern of symptoms that could be easily detected with the use of VR or an integrated assessment. Our purpose was to provide a heuristic decision tree for a more precise but simple diagnosis, giving evidence-based indications for a possible rehabilitation protocol using virtual reality tailored to these patients. The assessment of the OCD disorder was based on a clinical interview, integrated with a questionnaire, as well as neuropsychological assessment in rare cases. This last aspect is important because it may highlight a particular pattern of functioning in OCD patients [38]. We tried providing indications of the most useful assessment tools for the analysis of these specific aspects.

## 2. Materials and Methods

### 2.1. Participants

The 58 participants consisted of 29 OCD patients (mean age: 33.07; SD: 9.91), diagnosed by a clinical psychologist or psychiatrist as meeting the DSM-5 [1] criteria for OCD, and 29 healthy and partially matched controls (mean age: 40.48; SD: 15.59). The mean value for years of education (y.o.e.) of patients was 12.03 (SD: 3.2) and that for the healthy control group was 12.03 (SD: 3.2). In the OCD group, there were 14 females and 15 males, and, in the healthy control group, there were 14 females and 15 males. In Table 1, the descriptive statistics of both groups are presented. In Table 2, a comparison between age and y.o.e. and between the cognitive level of control and patient groups is presented. According to the results, the two groups were matched for both age and y.o.e. but not for cognitive level. The general exclusion criteria were as follows: (1) presence of sensory and/or motor limitation; (2) presence of deficit in general cognitive level (Mini Mental State Examination <19); (3) deficit in perception (Street Test <2.25); (4) deficit in language comprehension (Token Test <26.5); (5) anxiety (State Trait Anxiety Inventory - STAI >40); (6) depression (Beck Depression Inventory >16). We did not control the level of OCD symptoms with quantitative methods; however, all patients were currently undergoing treatment and, according to a clinician, in partial remission. Furthermore, OCD patients with comorbidities and healthy controls with any psychiatric diagnosis were excluded. The subjects involved were treated with both drugs and psychotherapy according to the standard. All participants were experienced with the use of personal computers (PCs) and came from the hospital’s area. Participants were asked not to drink caffeine or alcohol and not to smoke prior to the experimental test to avoid any effects of these substances on test execution and performance.

### 2.2. Ethics Statement

The study was approved by the scientific review board of the “U.O. di Psichiatria dell’Azienda Universitaria Ospedaliera Policlinico ‘Paolo Giaccone’ di Palermo”, in accordance with the Declaration of Helsinki. All participants gave written informed consent to the experimental procedure according to the rules of the scientific review board. All participant data were stored in encrypted and password protected files, following the criteria to protect personal health information [39].

### 2.3. Protocol

Participants were selected from the outpatient Unit of Psychiatry of Palermo University Hospital. The subject who met the experimental criteria were contacted, and a meeting was scheduled at the University Hospital. The experimental session was held by a specialized psychiatrist of the University of Palermo. At the beginning of the session, the examiner explained the general goals of the clinical protocol and the procedures to be used, and discussed the patient’s doubts and concerns. During the experimental session, two parts were planned: the classical neuropsychological assessment and the VR-based assessment. The presentation order was counterbalanced; half of the patients started the assessment with the VR test, and the other half started with the classic neuropsychological battery. Before the VR-based assessment, patients were trained for the use of a joypad within a virtual environment.

### 2.4. Neuropsychological Battery

To understand the cognitive profile of the participants, a complete neuropsychological battery was administered. A Mini Mental State Evaluation (MMSE) [40] was administered to assess the general cognitive level. To assess verbal memory, the Digit Span (Digit S) Test [41] was used to assess short-term memory, the Short Story Recall Test and the Paired-Associate Learning Test (PALT) [42] were used to assess long-term memory, and the Corsi Span (Corsi S) and the Corsi Block Task (Corsi BT) [41] were used for the assessment of short- and long-term spatial memory. For analysis of the executive domain, several tests were used: the Frontal Assessment Battery (FAB) [43], a general battery to assess frontal lobe functions, the Trail Making Test (TMT, forms A, B, and B-A) [44] for the assessment of selective attention, and the Tower of London (TOL) Test [45] for the assessment of planning abilities. Also, a Phonemic Fluency (PF) Test and a Semantic Fluency (SF) Test [46] were used. All scores of the tests were corrected for age, education level, and gender where appropriate.

### 2.5. VMET

The assessment protocol was created with NeuroVR (Version 2.0, Istituto Auxologico Italiano, Milan, Italy), a free software where the user can modify a pre-existing virtual environment by selecting contents from a database of objects (both two- and three-dimensional (2D and 3D)) and videos [47], expanded with NeuroVirtual 3D [48]. The scene was visualized in the player using non-immersive displays. The task took place in a virtual supermarket shown on a laptop screen, and the patient had to use a joypad to move around the environment. All users were trained for virtual reality use in another smaller shop, specifically designed for training purposes. In the virtual supermarket, all products were organized in categories such as beverages, fruits and vegetables, breakfast foods, hygiene products, frozen foods, garden products, and animal products.

### 2.6. VMET Scoring

Before starting the task, the participants received a shopping list, a sheet with the rules, a map of the supermarket, information about the supermarket (opening and closing times, products on sale, etc.), a pen, and a wristwatch. The examiner read and explained all the information relative to the subject in order to guarantee complete understanding. The VMET test was composed of four main tasks. The first involved purchasing six items (e.g., one product on sale). The second involved asking the examiner information about one item to be purchased. The third involved writing the shopping list 5 min after beginning the test. The fourth involved responding to some questions at the end of the virtual session by using the given materials (e.g., the closing time of the virtual supermarket). The rules that the patients had to follow to complete the task were as follows: (1) they had to execute all the proposed tasks; (2) they could execute all tasks in any order; (3) they could not go to a place unless it was a part of a task; (4) they could not pass through the same passage more than once; (5) they could not buy more than two items per category (looking at the chart); (6) they had to take as little time as possible to complete the exercise; (7) they could not talk to the researcher unless this was a part of the task; (8) they had to go to their “shopping cart” 5 min after the beginning of the task and make a list of all their products. After the explanation of the material, the clinician measured the time, stopping it when the participant said they finished the task. During the assessment, the examiner recorded all the participant’s behaviors in the virtual environment according to a predefined form. To better understand the patient’s work, the following items were recorded [49]: task failures (total and partial), inefficiencies, strategies, rule breaks, and interpretation failures. When a subtask was not totally completed, a task failure occurred, and the scoring range for total errors was from 11 (all 11 subtasks were correctly done) to 33 (all 11 subtasks were incorrectly done). To calculate the scoring for each task, the scale ranged from 1–3 (1 = the task was performed correctly; 2 = the participant performed part of the task; 3 = the participant totally omitted the task). An inefficiency was deemed a behavior that could prevent the correct execution of the tasks, such as not grouping similar tasks when possible. The general scoring range was from eight (several inefficiencies) to 32 (no inefficiencies), and the scoring scale for each inefficiency was from 1–4 (1 = always; 2 = more than once; 3 = once; 4 = never). To analyze the strategies, 13 behaviors that facilitated carrying out the tasks were evaluated, such as accurate planning before starting a specific subtask. The scoring scale for each strategy was from 1–4 (1 = always; 2 = more than once; 3 = once; 4 = never), and the total score ranged from 13 (good strategies) to 52 (no strategies). A rule break occurred when patients violated one or more of the eight rules listed (e.g., talking with the examiner when not necessary). The scoring scale for each rule break was from 1–4 (1 = always; 2 = more than once; 3 = once; 4 = never) and the total score ranged from eight (a large number of rule breaks) to 32 (no rule breaks). Finally, an interpretation failure occurred when the requirements of particular tasks were misunderstood, for example, when a participant thought that the subtasks all had to be done in the order presented on the information sheet. The general score ranged from three (a large number of interpretation failures) to six (no interpretation failures), and the score for each interpretation failure ranged from 1–2 (1 = yes; 2 = no). Furthermore, for every subtask, we analyzed the following variables: (1) sustained attention; (2) maintaining the correct sequence of the task; (3) remembering the instructions; (4) divided attention; (5) correct organization of the materials; (6) self-corrections; (7) absence of perseverations. The general score ranged from seven (no errors) to 14 (a large number of errors), and the score for each interpretation failure ranged from 1–2 (1 = yes; 2 = no). According to the analysis prosed by Cipresso and colleagues [35], we analyzed three subtasks that they recognized as particularly crucial in the OCD patients’ performance: (1) “going to the shopping chart after 5 min”; (2) “buying two products instead of just one”; (3) “going into a specific place and asking the examiner what to buy”. These tasks represented a break during the normal task execution because they required a different, confusing, or stopping behavior, which required attention and the elaboration of different information at the same time. These tasks represented a “break in time”, a “break in choice”, and a “break in social rules”, respectively.

### 2.7. Data Analysis

Data were analyzed with the aid of the statistical software STATA MP-Parallel Edition (Release 14.0, StataCorp LP, College Station, Texas) Orange (Version 3.3.5, Universitas Labacensis, Ljubljana and Portorož, Slovenia) with Python (Version 3.4, Python Software Foundation, Beaverton, OR, USA), JASP (Version 0.7.1.4, University of Amsterdam, Nieuwe Achtergracht, Amsterdam, The Netherlands) [50]. Comparisons between patients and controls were done by using a series of independent sample *t*-tests. To classify data, we used the following approaches [51,52]:

- Logistic regression classification algorithm with ridge regularization;
- Random forest classification using an ensemble of decision trees;
- Support vector machine (SVM), to map inputs to higher-dimensional feature spaces that best separated different classes.

Specifications about the algorithms used for computational data analyses can be found in the seminal article recently published by Zhou and colleagues (https://bmcmedinformdecismak.biomedcentral.com/track/pdf/10.1186/s12911-019-0890-0)

## 3. Results

Table 3 shows the results of OCD patients compared with normative data. The results showed intact cognitive levels in these patients.

Table 4 and Table 5 report the sample descriptive statistics for the neuropsychological battery and the VMET scores, respectively. Table 6 indicates the independent sample *t*-tests comparing OCD patients with controls, for both the executive function domain and the other cognitive domains. On the other hand, Table 7 reports the independent sample *t*-tests for the VMET scoring.

Clearly, both the neuropsychological battery and the VMET scores were able to differentiate patients from healthy controls; however, the mean scores of the neuropsychological battery for both patients and healthy controls were situated in the normal range (Table 3). Because of this fact, it is important to define classification models able to identify mutual information among the variables to make predictions based on a limited number of tests in a clinical setting. To pursue this aim, we ran three different learning algorithms for a cross-validation based on logistic regression, random forest, and support vector machine. Two different models were built: one based on classical neuropsychological tests for executive functions (FAB, TMTA, TMTB, TMTBA, TOL, PF, and SF) and the other one by also adding the previously defined VMET scores. Results of the two cross-validations can be seen in Table 8.

**Figure 1:**
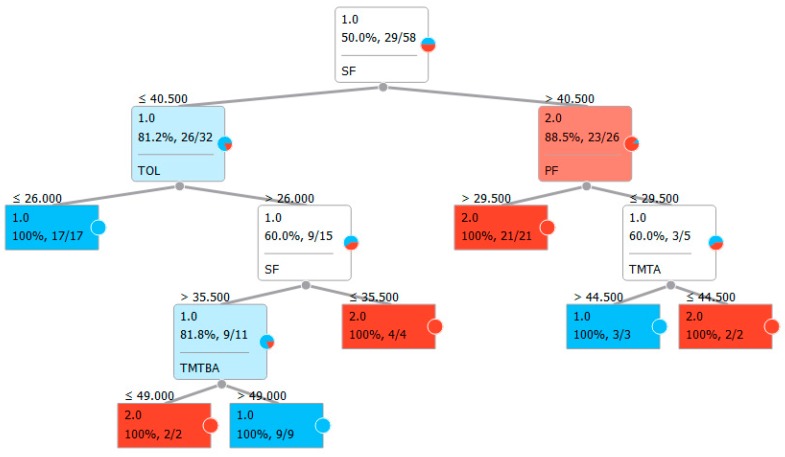
Classification tree using classic neuropsychological test for executive functions.

**Figure 2:**
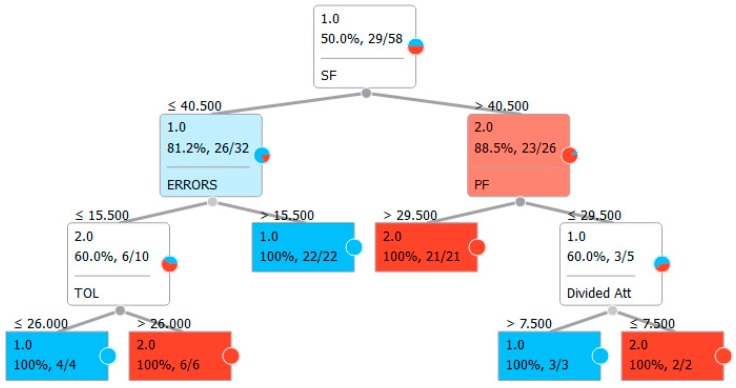
Classification tree using the classic test for executive functions and the virtual multiple errands test (VMET).

Finally, a classification tree for both models was built based on feature selection, choosing entropy as a measure of homogeneity [56,57,58] for split selection (Figure 1 and Figure 2). Small circles indicate the ratio of classifications reported inside the rectangle in terms of percentage of correctness in recognizing the specific characteristics. The colors indicate classification as one of the two groups: blue for OCD patients, red for control participants.

## 4. Discussion

The general aim of this study was twofold. Firstly, we investigated executive functions in OCD patients and controls. To this purpose, we used the virtual version of the multiple errands test and a classic neuropsychological battery. On the other hand, our purpose was to find a method to discriminate the two groups in a better way. Indeed, the goal was scaled on the minimal number of variables possible to pursue an ecological assessment of executive functions with these patients. VMET was demonstrated to be effective in the assessment of several patients, such as for OCD [35], Parkinson’s disease [28], and stroke [49]; however, previous studies focused on single scores, such as time, total and partial errors, inefficiencies, and others. In this study, we exploited the multivariate nature of the VMET for the assessment of a particular patient sample (i.e., OCD), where dysfunction is slightly higher than or equal to a normative sample. Thus, this aim shed new light on using multidimensional scaling for understanding the deficits of executive functions.

The results showed a clear difference between OCD patients and the control group, particularly in executive functions, as highlighted in Table 4 for the classic neuropsychological test and Table 5 for most of the VMET scores. However, the complexity of a complete neuropsychological battery with the complete execution of VMET hindered the assessment of these patients. With this limitation in mind, we used computational techniques, which are also used in VR settings [59], to advance our knowledge based on consistent and relevant data from the sample presented. The first strategy was to understand the classification ability within the sample with a supervised machine learning approach. The results showed precision levels between 71.4% and 84.6%, making us confident of the goodness of fit of the model to data. This result is important since, on the one hand, it allowed discriminating OCD patients from healthy subjects (see Table 6, where results are all related to such a domain). On the other hand, at the clinical level, we need to reduce complexity and, consequently, the number of used tests. To this purpose, we opted for a visual classification tree to provide clear indications (based on data) of heuristic choice (Figure 1 and Figure 2). The main and most encouraging result was that, by using both the classic neuropsychological battery and the VMET scores, we obtained a tree based on only five variables (Figure 2), two of which are VMET-based and, not surprisingly, particularly related to OCD patients (errors and divided attention).

Even if the tree based on classic neuropsychological battery (Figure 1) could provide a useful tool for patient evaluation, it does not represent a simple tool to be used for the assessment of executive functions as a whole. On the other hand, the tree based on semantic and phonemic fluency, as well as the TOL and the two VMET scores (Figure 2), can be very useful as a shortened model for the assessment of executive functions in OCD patients.

During the assessment process of OCD, the clinician has many variables to take into account, and deficits of executive function could be one of them. A method which provides clinicians with a simple and complete indication of the best assessment process may have great impact on the clinical process and on rehabilitation.

In clinical neuropsychological assessment, there is always the necessity of integrating different types of information, such as psychometric and ecological data [60,61] in order to better understand the patient’s cognitive functioning. VMET is able to play a crucial role in integrating classical neuropsychological tests with ecological settings, especially for executive function assessment in different type of patients.

Making a guided diagnosis for a specific cognitive domain in a specific target of patients is an important goal for both the assessment and the rehabilitation process because it could be able, on one hand, to reduce the time and effort expended by patients and clinicians. On the other hand, a virtual rehabilitation program developed for a targeting assessment would potentially be more personalized and efficient.

This study also had some limitations that could be overcome in future studies. Firstly, the sample size was limited; in a future study, an accurate sample size calculation could be done. Also, adding a clinical control group could be interesting in order to understand the potential of our algorithm for differential diagnosis.

According to our algorithm, the important test for discrimination between OCD patients and controls are fluency, both semantic and phonological, Tower of London, and VMET. This setting would require a minimum amount of time and reduced effort for the clinician during the assessment procedure.

## Figures and Tables

**Table 1:** Descriptive statistics of the two groups. SE—standard error; y.o.e.—years of education; MMSE—Mini Mental State Examination.

	Group	*N*	Mean	SD	SE
**Age**	1	29	33.07	9.906	1.840
	2	29	40.48	15.588	2.895
**y.o.e.**	1	29	12.03	3.201	0.594
	2	29	12.03	3.029	0.563
**MMSE**	1	29	26.56	2.675	0.497
	2	29	28.53	1.028	0.191

**Table 2:** Independent sample Mann–Whitney U test.

	W	*p*
**Age**	312.5	0.094
**y.o.e.**	425.5	0.936
**MMSE**	194.0	<0.001

**Table 3:** Neuropsychological battery in obsessive–compulsive disorder (OCD) patients compared to deficit level in normative sample.

Test	Mean	Standard Deviation	Normative Data
MMSE	26.56	2.68	>18
Frontal Assessment Battery (FAB)	14.97	1.4	>13.5
Trail Making Task A (TMTA)	63.07	23.58	<93
Trail Making Task B (TMTB)	191.93	112.04	<282
Trail Making Task B-A (TMTBA)	129.93	100.27	<186
Phonemic Fluency (PF)	27.38	9.42	>16
Semantic Fluency (SF)	33.69	8.43	>24
Tower of London (TOL)	22.72 ^a^	5.45	Not available
Digit Span (Digit S)	5.28	1.09	>3.5
Paired-Associate Learning Test (PALT)	10.84	4.04	>6
Corsi Span (Corsi S)	4.51	0.78	>3.5
Short Story	12.62	5.24	>7.5
Corsi Block Task (Corsi BT)	16.09	8.21	>5.5

^a^ Normally considered non-pathological level.

**Table 4:** Classical neuropsychological battery descriptive statistics (Group 1: OCD patients; Group 2: controls).

Test	Group	*N*	Mean	SD	SE
MMSE	1	29	26.565	2.675	0.497
	2	29	28.532	1.028	0.191
FAB	1	29	14.965	1.403	0.261
	2	20	16.274	0.849	0.190
TMTA	1	29	63.069	23.584	4.379
	2	29	37.632	15.624	2.901
TMTB	1	29	191.310	112.041	20.806
	2	29	95.448	46.144	8.569
TMTBA	1	29	129.931	100.269	18.619
	2	29	58.616	45.439	8.438
PF	1	29	27.379	9.420	1.749
	2	29	41.138	11.192	2.078
SF	1	29	33.690	8.431	1.566
	2	29	48.172	10.275	1.908
TOL	1	29	22.724	5.450	1.012
	2	29	28.448	3.582	0.665
Digit S	1	29	5.284	1.087	0.202
	2	29	6.010	0.847	0.157
PALT	1	29	10.845	4.036	0.749
	2	20	13.072	4.759	1.064
Corsi S	1	29	4.508	0.778	0.144
	2	29	6.345	2.660	0.494
Short Story	1	29	12.621	5.242	0.973
	2	29	14.491	4.454	0.827
Corsi BT	1	29	16.091	8.208	1.524
	2	29	21.239	5.843	1.085

**Table 5:** Virtual multiple errands test (VMET) descriptive analysis.

VMET	Group	*N*	Mean	SD	SE
Errors	1	29	17.276	2.840	0.527
	2	29	13.897	1.633	0.303
Break in time	1	29	13.379	2.821	0.524
	2	29	11.655	2.844	0.528
Break in choice	1	29	9.655	1.951	0.362
	2	29	8.379	0.979	0.182
Break in social rules	1	29	10.517	2.181	0.405
	2	29	8.793	1.544	0.287
Inefficiencies	1	29	22.552	4.733	0.879
	2	29	24.379	6.439	1.196
Rule break	1	29	21.172	3.733	0.693
	2	29	22.897	5.453	1.013
Strategies	1	29	36.414	7.238	1.344
	2	29	31.793	6.298	1.170
Interpretation failures	1	29	5.207	0.940	0.175
	2	29	5.241	0.872	0.162
Time	1	29	649.448	320.076	59.437
	2	29	595.759	266.793	49.542
Sustained attention	1	29	8.345	1.610	0.299
	2	29	7.759	0.830	0.154
Sequence	1	29	8.241	1.640	0.305
	2	29	7.828	0.805	0.149
Instructions	1	29	8.276	1.623	0.301
	2	29	7.517	0.634	0.118
Divided attention	1	29	10.448	2.667	0.495
	2	29	8.276	1.556	0.289
Organization	1	29	10.483	3.158	0.586
	2	29	8.000	1.282	0.238
Self-corrections	1	29	9.241	1.902	0.353
	2	29	7.759	0.786	0.146
Perseverations	1	29	8.724	1.830	0.340
	2	29	7.414	0.682	0.127

**Table 6:** Independent sample *t*-Test comparing OCD patients vs. controls for neuropsychological battery. df—degrees of freedom.

Test	*t*	df	*p*	Mean Difference	SE Difference	Cohen’s *d*
**Executive Function Domain** **↓**						
FAB	−4.061	46.36	<0.001	−1.309	0.322	−1.082
TMTA	4.842	48.61	<0.001	25.437	5.253	1.272
TMTB	4.260	37.23	<0.001	95.862	22.501	1.119
TMTBA	3.489	39.04	0.001	71.315	20.442	0.916
PF	−5.065	54.42	<0.001	−13.759	2.717	−1.330
SF	−5.868	53.94	<0.001	−14.483	2.468	−1.541
TOL	−4.726	48.38	<0.001	−5.724	1.211	−1.241
**Other Cognitive Domains** **↓**						
MMSE	−3.696	36.10	<0.001	−1.967	0.532	−0.971
Digit S	−2.836	52.84	0.006	−0.726	0.256	−0.745
PALT	−1.712	36.44	0.095	−2.228	1.301	−0.513
Corsi S	−3.568	32.75	0.001	−1.837	0.515	−0.937
Short Story	−1.465	54.58	0.149	−1.871	1.277	−0.385
Corsi BT	−2.752	50.58	0.008	−5.149	1.871	−0.723

*Note.* For all tests, variances of groups were not assumed equal.

**Table 7:** Independent sample *t*-test for VMET index.

VMET	*t*	df	*p*	Mean Difference	SE Difference	Cohen’s *d*
**Errors**	**5.555**	**56.00**	**<0.001**	3.379	0.608	1.459
Break in time	2.318	56.00	0.024	1.724	0.744	0.609
Break in choice	3.148	56.00	0.003	1.276	0.405	0.827
Break in social rules	3.474	56.00	<0.001	1.724	0.496	0.912
Inefficiencies	−1.232	56.00	0.223	−1.828	1.484	−0.323
Rule break	−1.405	56.00	0.166	−1.724	1.227	−0.369
Strategies	2.593	56.00	0.012	4.621	1.782	0.681
Interpretation failures	−0.145	56.00	0.885	−0.034	0.238	−0.038
Time	−0.033	56.00	0.974	−2.117	64.666	−0.009
Sustained attention	1.743	56.00	0.087	0.586	0.336	0.458
Sequence	1.220	56.00	0.228	0.414	0.339	0.320
Instructions	2.344	56.00	0.023	0.759	0.324	0.616
Divided attention	3.789	56.00	<0.001	2.172	0.573	0.995
Organization	3.923	56.00	<0.001	2.483	0.633	1.030
Self-corrections	3.879	56.00	<0.001	1.483	0.382	1.019
Perseverations	3.613	56.00	<0.001	1.310	0.363	0.949

*Note.* For all tests, variances of groups were assumed equal.

**Table 8:** Classification table. Three learning algorithms were compared, namely, logistic regression (LogReg), random forest, and support vector machine (SVM). In the first analysis (Panel A), the classifications were run referring to the classic neuropsychological test used for executive functions. In the second analysis (Panel B), VMET scores were added to the classic test for the classification learning algorithm [53,54,55].

**Panel A: Classification with classic neuropsychological test for executive functions.**					
**Features:**	*FAB, TMTA, TMTB, TMTBA, TOL, PF, SF*				
**Sampling type:**	Stratified 10-fold cross-validation				
**Target class:**	Average over classes				
**Method**	**AUC**	**CA**	**F1**	**Precision**	**Recall**
LogReg	0.742	0.741	0.746	0.733	0.759
Random forest	0.817	0.810	0.800	0.846	0.759
SVM	0.783	0.776	0.787	0.750	0.828
**Panel B: Classification with classic neuropsychological test for executive functions and the VMET.**					
**Features**	*FAB, TMTB, TMTA, TMTBA, TOL, PF, SF, ERRORS, break in time, break in choice, break in social rules, inefficiencies, rule break, strategies, interpretation failures, sustained attention, sequence, instructions, divided attention, organization, self-corrections, perseverations*				
**Sampling type**	Stratified 10-fold cross-validation				
**Target class**	Average over classes				
**Method**	**AUC**	**CA**	**F1**	**Precision**	**Recall**
LogReg	0.700	0.707	0.702	0.714	0.690
Random forest	0.850	0.845	0.852	0.812	0.897
SVM	0.775	0.776	0.772	0.786	0.759

AUC (area under the receiver operating characteristic (ROC) curve) is the area under the classic receiver operating characteristic curve. CA (classification accuracy) represents the proportion of examples correctly classified. F1 represents the weighted harmonic average of precision and recall (see below). Precision represents the proportion of true positives among all instances classified as positive. In our case, this was the proportion of OCD patients correctly identified as patients and not controls. Recall represents the proportion of true positives among the positive instances in our data, i.e., the number of OCD patients diagnosed as patients instead of controls.
